# Role of viruses in the development of breast cancer

**DOI:** 10.1186/1750-9378-8-32

**Published:** 2013-09-02

**Authors:** Kenneth Alibek, Ainur Kakpenova, Assel Mussabekova, Marzhan Sypabekova, Nargis Karatayeva

**Affiliations:** 1 Nazarbayev University, 53 Kabanbay Batyr Avenue, Astana 010000, Kazakhstan; 2 National Medical Holding, 2 Syganak Street, Astana 010000, Kazakhstan

**Keywords:** Carcinogenesis, Infectious agents, Breast cancer

## Abstract

The most common cancer worldwide among women is breast cancer. The initiation, promotion, and progression of this cancer result from both internal and external factors. The International Agency for Research on Cancer stated that 18-20% of cancers are linked to infection, and the list of definite, probable, and possible carcinogenic agents is growing each year. Among them, biological carcinogens play a significant role. In this review, data covering infection-associated breast and lung cancers are discussed and presented as possible involvements as pathogens in cancer. Because carcinogenesis is a multistep process with several contributing factors, we evaluated to what extent infection is significant, and concluded that members of the herpesvirus, polyomavirus, papillomavirus, and retrovirus families definitely associate with breast cancer. Detailed studies of viral mechanisms support this conclusion, but have presented problems with experimental settings. It is apparent that more effort needs to be devoted to assessing the role of these viruses in carcinogenesis, by characterizing additional confounding and synergistic effects of carcinogenic factors. We propose that preventing and treating infections may possibly stop or even eliminate certain types of cancers.

## Introduction

Globally, the most frequent cancer among women is breast cancer [1]. As a result of this prevalence, this cancer constitutes the highest number of deaths, involving 458,000 deaths per year, with the number of cases continuing to rise [2].

The development of breast cancer occurs as a result of numerous internal and external factors. Carcinogenesis of breast cancer has been associated with genetic predisposition (e.g., mutations in BRCA1/2 and other genes), a family history of breast cancer, ethnicity (more common in the Caucasian population), dense breast tissue, lifestyle, hormonal contraception and treatment after menopause, and obesity [2].

External factors also play major roles during initiation, development, and progression of cancer. The International Agency for Research on Cancer (IARC) reports that biological carcinogens cause 18-20% of cancers [3]. Recently, the roles of infections during carcinogenesis in several types of oncological diseases were comprehensively summarised [4-6]. We therefore reviewed numerous studies in an attempt to determine whether certain viruses might be implicated in breast cancer, and we show association of this cancer with herpesvirus, polyomavirus, papillomavirus, and retrovirus. Several mechanisms of tumourigenic action of infectious agents have been proposed including NF-κB, STAT3, HIF1α pathways, and a possible role of cofactors [7-11]. Nevertheless, data on viral presence and oncogenic mechanisms reported in the recent literature are still inconsistent, and detailed mechanisms of interactions between infectious agents and host cells have yet to be fully elucidated.

### Herpesvirus: human cytomegalovirus (HCMV) and Epstein-Barr virus (EBV)

Human herpesvirus is known for its oncogenic potential. HCMV and EBV of the *Herpesviridae* family have been implicated as a cause of breast cancer. EBV is classified as a class I carcinogen by IARC [5]. Analysis of EBV associations with breast cancer risk shows contradictory results [12]. It is believed that the contradictions arise from the different methodologies used [13,14]. In polymerase chain reaction (PCR)-based studies, positive correlation was shown [12], and the presence of EBV DNA was associated with more severe forms of breast cancer [15]. In contrast, studies using EBV encoded RNA *in situ* hybridisation (EBER-ISH) have shown negative correlations even when PCR results were positive [13]. In a recent study, EBER-ISH results showed the presence of EBV in 47.5% of cases. However, the virus was localised to infiltrating lymphocytes in the tumour microenvironment rather than in tumour cells [13]. This may suggest that causal association of the EBV and breast cancer might be due to changes in viral expression, resulting in changes in the tumour microenvironment. Consistent with this possibility, an increase in IgA antibodies against EBV viral capsid antigen and nuclear antigen-1 (EBNA-1) was positively associated with breast cancer risk. Moreover, the mechanism of EBNA-1 action can be associated with the genetic polymorphisms of interferon-γ [16,17]. Lastly, statistical association of the virus with increased breast carcinoma risk was shown after recent analysis of 1535 cases [18].

HCMV has been shown to be involved in many cancers including malignant glioma, and prostate, skin, and colorectal cancers. Breast milk is one of the main routes of transmission of the virus, and breast epithelium may therefore be a major site of latent and active HCMV infection. When using immunohistochemistry to compare biopsy specimens from breast cancer patients versus controls, it was shown that HCMV infection may occur in breast epithelium of both normal and cancer patients. However, infection had a much higher rate (97%) in breast carcinoma cases [19]. There is also an association between elevation of serum CMV IgG levels and breast cancer [20]. Analysis of IgG GM allotypes, which are associated with certain tumour antigens, showed that the most relevant non-self antigen in breast cancer patients was HCMV. It was speculated that the presence of a certain allotype and seropositivity for HCMV could have cumulative effects leading to breast cancer development [21]. However, CMV was not detected by recent RT-PCR analysis when investigating the prevalence of viruses in breast cancer tissue [22,23].

There might be several mechanisms of how CMV can cause breast cancer initiation and progression. Firstly, it was shown that HCMV gene products affect cell cycle regulation, inhibit apoptosis, activate angiogenesis and metastatic phenotype, and cause increased mutation rate, thereby overlapping with all established hallmarks of cancer cells [24]. Secondly, HCMV exhibits immunosuppressive properties, leading to escape of tumour cells from immune surveillance mechanisms [24,25]. Thirdly, specific actions of virus-encoded interleukins (IL) can be implicated. In a recent review [26] the role of IL-10 in breast cancer is discussed. Interestingly, both breast tumour inhibiting and promoting effects of the cytokine were shown. IL-10 was shown to be differentially expressed in breast tumour cells and infiltrating lymphocytes. Elevated serum level of IL-10 was observed in breast cancer patients. The fact that HCMV expresses a viral analogue of human IL-10 may lead to the conclusion that this could be one of the mechanisms of breast cancer promotion by the virus.

### Polyomavirus: SV40 and John Cunningham virus (JCV)

Members of the polyomavirus family, such as SV40, JCV, BK, and Merkel cell polyomavirus (MCPyV) have been associated with oncogenic diseases. Recently, 54 fresh frozen breast tumour samples were analyzed for the presence of 10 polyomaviruses [22]. The results showed no detection of JCV and BK in the invasive ductal type carcinomas, as opposed to previous reports [27]. This difference in results could be due to differing laboratory techniques and/or small sample size. Interestingly, a related BK virus (BKV), in the same case study, gave negative results by direct sequencing [27]. Breast cancer samples were also negative for MCPyV [28]. Although, the presence of polyomaviruses within a tumour sample can show a link to tumour formation, it is important to identify viral mechanisms that lead to carcinogenesis. Below, we discuss results showing that polyomaviruses can directly cause cell immortalisation, hypermethylation of tumour suppressors, and spontaneous genetic alternations. Thus, they play an important role in breast cancer.

SV40 virus was introduced into humans in the 1950s, when contaminated poliovirus vaccine was injected into millions of people. Since then, the involvement of SV40 in various cancers (e.g., brain, lung, colon, breast, and prostate) has been controversial [29]. Such cancers contained SV40 DNA, which was mostly detected by PCR [30-32]. The SV40 presence was investigated by PCR which targeted Tag in 109 breast carcinomas, showing that SV40 DNA sequences were found in 22% of cancers. Immunohistochemical analyses confirmed the presence of virus in cancer cells.

Development of breast cancer within normal epithelial cells has been studied in mice, and has been related to sequential changes in a permissive microenvironment [33]. Mice and rat models showed that SV40 T/t-antigen expression in mammary epithelium results in pre-neoplastic lesions that progress to invasive and metastatic cancers [34,35]. Moreover, the immortalisation of normal human mammary epithelial cells can be achieved by SV40-induced transformation. SV40 large T-antigen contains an Rb-binding domain which causes altered gene expression and loss of p16(INK4a) expression [36]. Lastly, RASSF1A, SHP1, BRCA1, and TIMP3 methylation was higher in SV40-positive cases, with higher levels of P53 protein [37].

Further investigations of JCV in tissues of breast cancer patients showed that JCV T-antigen DNA was detected in 23% of breast carcinoma tissues [27]. JCV T-antigen binds and affects wild type p53, stabilizes b-catenin, and causes chromosomal instability. It activates ATM- and ATR-mediated G2 checkpoint pathways and causes G2 cell cycle arrest [38]. Expression of this protein also causes a metastatic phenotype in colorectal cancers [39].

### Papillomavirus: HPV

HPV is a small DNA virus that is more often associated with cervical cancer in women. High-risk HPV types 16 and 18 have been implicated in 70% of all cases of cervical cancer [40]. HPV was also found in anogenital and oral carcinomas. Based on this evidence, HPV was classified as an oncovirus by IARC. HPV usually infects keratinocytes and mucous membranes. The virus is able to integrate itself into the host cell genome and use its transcription machinery to express viral proteins. Two of these proteins, early protein 6 and 7 (i.e., E6 and E7) inactivate tumour suppressor proteins p53 and pRb, respectively, and early protein 5 (E5) can affect receptor tyrosine kinases by associating with the cell membrane [41].

There is considerable controversy regarding the role of HPV in breast cancer. A recent control study summarised the results of molecular studies on the detection of high risk HPV in breast cancer samples and concluded that positive associations ranged from 0 to 86% [42]. The majority of molecular studies employed standard or nested PCR as methods of detection using commercially available primers for the L1 gene (codes for capsid protein). However, after obtaining positive results, primers for E6 and E7 genes, or for sequencing, reported possible sources of false positive and false negative results and limitation factors associated with detection methods used in those studies, such as confirmation of DNA/RNA quality, and adjustment for confounding factors [43]. Hernandez et al. reported lack of L1 expression in invasive anal and cervical carcinomas using immunohistochemical evaluation [44]. This might also be the case with advanced breast cancer samples. It is also possible that the extent of HPV implicated in breast cancer is higher than studies have reported. Moreover, it has been reported recently that HPV is present in human breast milk [45], which indicates that the virus indeed can infect breast tissue and accumulate in it. Possible mechanisms of HPV in breast cancer carcinogenesis could be the same as in anogenital and head and neck tumours, via E6 and E7, or through a different pathway. High-risk HPV infection was associated with upregulated expression of the Id-1 transcription factor (a family of helix–loop–helix transcription factors) in aggressive breast cancer tissues, and suggested that the virus can induce cell invasion and metastasis via Id-1 [46,47]. Consistent with this possibility, Frega et al. did not observe expression of E6 and E7 in HPV-positive breast cancer tissues [48]. However, Dimri et al. were able to immortalise human mammary epithelial cells in culture using E6 and E7 oncogenes [49].

Overexpression of c-MYC gene is a signature of the majority of breast cancers [50], and there is a significant association between elevated levels of c-Myc and HPV 16 infection. It was also shown that HPV 18 integrates in the proximity of c-myc in cervical carcinoma [51], and that there is HPV-mediated activation of c-Myc. E6 was not only found to elevate levels of c-Myc, but also was able to associate with the Myc complex and drive expression of its target genes. Moreover, c-Myc induces TERT (telomerase reverse transcriptase) transcription in the presence of E6 [52], which leads to increased telomerase activity, and hence immortalises HPV-infected cells. E7 was also found to elevate levels of c-Myc. This was observed in murine C127 cells infected with bovine papillomavirus type 1 [53] and in cells expressing HPV E7 [54,55]. Wang et al. [56] were able to determine that E7 enhances c-Myc binding to the hTERT promoter, and suggested that E6 and E7 may work synergistically in order to induce transcription from the TERT promoter.

There is also a possibility that latent viruses can be activated by sex hormones. Aceto et al. [57] detected HPV 16 in two cases of juvenile breast cancer after menarche, and one case of breast cancer after pregnancy and lactation. One case of juvenile breast cancer was positive for both HPV 16 and HPV 18. They were also able to detect E6 in peripheral blood samples from patients with stage IV juvenile breast cancer [57]. The possibility of this DNA fragment coming from latent urogenital infection was excluded because this was only the case in advanced cervical carcinoma. This is consistent with the finding that steroid hormones are able to bind to several regions in the control region of the HPV genome (LCR), enhancing expression of E6 and E7 of high-risk HPVs [58].

### Beta retrovirus: human mammary tumour virus (HMTV)

Investigators have tried to determine the involvement of HMTV in human breast cancers since 1943, when mouse mammary tumour virus (MMTV) was shown to cause mammary cancers in mice [59]. Several groups established that MMTV-like sequences were present in human breast cancer samples, but absent in normal tissues [60]. Furthermore, HMTV isolated from primary cultures of breast cancer cells had 95% homology with MMTV. In mice, the HMTV homolog promotes tumour formation through the insertion mutagenesis of Wnt oncogenes, thereby promoting its activation. In a recent study, Wnt-1 expression was higher in specimens which were positive for *env*, an envelope protein of MMTV, compared with *env*-negative specimens [61]. *Env* is typically absent in normal tissues and present in breast cancer tissues in both mice and human. In addition to the Wnt region, the common integration sites for MMTV were found to be in loci 35 that contain regions of the Fgf and Rspo gene families. The sites were frequently activated in tumours induced by MMTV, which primarily caused mammary premalignant hyperplastic outgrowth. Other genes were also deregulated, including Phf19 and Fox1. For example, Phf19 increased cell invasion capability and Fox1 promoted anchorage independent colony formation of MMTV infected cells. Approximately 20 of the HMTV common insertion site-associated genes are deregulated and/or mutated in human breast tumours [62]. HMTV sequences for *env*, *gag*, and *sag* from patients with ductal carcinoma and mammary hyperplasia have been cloned and sequenced, revealing the viral gene existence. The study hypothesised that the expression of HMTV sequences could be a risk factor for genome instability and for disease development [63]. Moreover, the localisation of the MMTV *env* sequences to the nuclei of human breast cancer cells indicated that the provirus integrated into cancer cells [61]. These MMTV viral sequences were more common in conditions such as gestational [64] and familial breast cancers, indicating that a provirus could be transmitted or inherited. In addition, geographic localisation might also play a role in virus distribution. For example, the highest prevalence of viral sequence-positive breast cancer was in Tunisia, a country known to have the highest prevalence of rapidly progressing inflammatory breast cancer in the world [65]. Inflammatory breast cancer is a form of breast cancer in which the presence of viral sequences are found to be associated with tumour aggressiveness in patients [66]. When the investigators examined samples from patients with both diseases, they found viral sequences in breast tissues as well as in lymphoma tissues.

Cells infected with MMTV, like many other cancer cells, have highly active Src kinase. MMTV expressing cells escape apoptosis by activation of immune receptor tyrosine kinase-based activation motif-mediated Src tyrosine kinase signalling pathways [67]. EBV (68%), HPV (50%) and MMTV (78%) gene sequences are present and co-exist in many human breast cancers. Normal controls showed these viruses were also present in epithelial cells in human milk with less occurrence compared to breast cancer cells. The presence of these viruses in breast cancer is associated with younger age of diagnosis and possibly an increased grade of breast cancer [68]. These findings provide further evidence for a potential role of HMTV in breast cancer, but needs to be further investigated.

## Conclusions

Carcinogenesis can involve several factors, including genetic predisposition, environment, and changes in the immune system. The role of viruses in most common cancers is certainly important, and in our opinion, highly underestimated. Viruses can act as direct transforming agents and as triggering cofactors. The most probable mechanism of carcinogenesis may involve a combination of genetic alterations, immune system dysfunctions, and viral infections [69].

The data reviewed in this article emphasises the importance of further studies, which could elucidate viral mechanisms that can lead to breast cancer. It is also important to acquire more clinical and epidemiological data on the combination of factors which might, together with infection, lead to cancer development. The possibility of including antiviral agents in breast cancer therapy should be considered, as they are in other infection-associated cancer types such as hepatocellular carcinoma, brain tumours, Kaposi sarcoma, nasopharyngeal carcinoma, and some hematopoietic cancers [70]. Further investigation of the role of viruses in breast cancer may result in new discoveries that could lead to better diagnosis, prevention, and treatment of these cancers.

## Abbreviations

HPV: Human papillomavirus; EBV: Epstein-Barr virus; HCV: Hepatitis C virus; HBV: Hepatitis B virus; HCMV: Human cytomegalovirus; MMTV: Mouse mammary tumour virus; SV40: Simian virus 40; Env protein: Envelope protein; BKV: BK virus.

## Competing interests

The authors declare that they have no competing interests.

## Authors’ contributions

KA, MS, AM, NK, and AK performed the literature research and wrote the manuscript. All authors have read and approved the final manuscript.
